# Parent-Teen Sexual Health Communication and Teens’ Health Information and Service Seeking

**DOI:** 10.1001/jamanetworkopen.2025.41712

**Published:** 2025-11-05

**Authors:** Hannah Javidi, Jorge V. Verlenden, Xiwei Chen, Eric R. Walsh-Buhi

**Affiliations:** 1 Department of Psychology, North Carolina A&T State University, Greensboro; 2 Center of Excellence for Integrative Health Disparities and Equity Research, North Carolina A&T State University, Greensboro; 3 Department of Applied Health Science, Indiana University School of Public Health–Bloomington, Bloomington; 4 Center for Sexual Health Promotion, Indiana University School of Public Health–Bloomington, Bloomington; 5 Centers for Disease Control and Prevention, National Center for Chronic Disease Prevention and Health Promotion, Division of Adolescent and School Health, Atlanta, Georgia; 6 Department of Epidemiology and Biostatistics, Indiana University School of Public Health–Bloomington, Bloomington; 7 Cancer Prevention and Control Research Program, Indiana University Melvin and Bren Simon Comprehensive Cancer Center, Indianapolis

## Abstract

**Importance:**

Parent-teen sexual health communication can support and empower teens in their sexual and reproductive health (SRH) decision-making. These conversations may differ in their associations with teen health outcomes across parent-teen dynamics and parents’ knowledge about and comfort with the topic.

**Objectives:**

To investigate parent and teen characteristics associated with the frequency of parent-teen sexual health communication, parent information adequacy, and parent communication comfort and the association between communication frequency and teens’ self-efficacy to seek SRH information and services and to examine the moderating effects of parents’ perceived information adequacy and communication comfort.

**Design, Setting, and Participants:**

This cross-sectional survey comprised a probability sample of 522 parent-teen (aged 15-17 years) dyads representative of the US household population from the 2022 Teen and Parent Surveys of Health. Participants were recruited from AmeriSpeak and AmeriSpeak Teen panels, administered by the National Opinion Research Center (University of Chicago) from May 12 to September 30, 2022. Statistical analysis was performed from February to May 2024.

**Main Outcomes and Measures:**

The frequency of parent-teen sexual health communication and teens’ self-efficacy to seek SRH information and services. Moderators were parent information adequacy and communication comfort. All analyses were weighted.

**Results:**

Of 522 parents, most were female (377 [61.3%]), 45 years of age or older (214 [53.5%]), White (309 [57.0%]), and had less than a bachelor’s degree (325 [56.7%]). Of 522 teens, half were male (244 [50.3%]), and most were heterosexual (394 [79.8%]). Female parents (54.5% [95% CI, 48.6%-60.2%]; *P* = .001), parents aged 18 to 44 years (58.0% [95% CI, 51.3%-64.4%]; *P* < .001), and Black parents (74.3% [95% CI, 56.5%-86.6%]; *P* = .01) communicated about sexual health most frequently. Parents with a Bachelor’s degree or higher (36.8% [95% CI, 30.0%-47.9%]; *P* = .03) and household incomes higher than $100 000 (31.1% [95% CI, 22.6%-41.1%]; *P* < .001) were significantly less likely to communicate about sexual health. Teens whose parents talked frequently had higher self-efficacy when parents were more informed (β = 0.11 [95% CI, 0.03-0.20]; *P* = .01) and more comfortable (β = 0.11 [95% CI, 0.01-0.20]; *P* = .03); however, if parents were not informed or comfortable, frequent communication was associated with lower teen self-efficacy.

**Conclusions and Relevance:**

In a cross-sectional, nationally representative survey of 522 parent-teen dyads, frequent parent-teen sexual health communication was associated with increased teen self-efficacy for SRH information and service seeking, but this depended on how comfortable and informed their parents felt. These findings suggest that parents must possess accurate information and comfort to discuss sexual health topics.

## Key Points

**Question:**

Is the frequency of parent-teen sexual health communication associated with teens’ self-efficacy to seek sexual and reproductive health (SRH) information and services?

**Findings:**

In this cross-sectional study of 522 parent-teen dyads, frequent communication was associated with higher teen self-efficacy when parents felt informed and comfortable. When parents did not feel informed or comfortable, frequent communication was associated with decreases in teen self-efficacy.

**Meaning:**

This study suggests that parents must have quality information and strategies to increase comfort around sexual health conversations, which may in turn be associated with higher levels of teens’ confidence to seek SRH information and services when needed.

## Teaser

This cross-sectional study examines the association of the frequency of parent-teen sexual health communication with teens’ self-efficacy to seek sexual and reproductive health information and services.

## Introduction

Parent-teen sexual health communication (ie, bidirectional communication between parents or parental figures and their teenage children about sex, sexuality, and sexual health outcomes)^1^ can support positive teen sexual health behaviors and outcomes.^2^ Findings from conceptual and meta-analytic reviews indicate that parent-teen sexual health communication is robustly related to teens’ safer-sex cognitions (eg, condom-related attitudes, self-efficacy to resist unwanted sexual encounters) and behaviors, including contraceptive and condom use.^3,4^

By fostering open, frequent dialogue around sex, parents can equip their teens with the knowledge and skills necessary to navigate complex sexual and reproductive health (SRH)–related issues.^5^ One goal of effective parent-teen communication is preparing adolescents for adulthood by empowering them with skills to proactively take their health into their own hands, including the ability to make informed decisions and seek health care when needed.^6,7,8,9^ Studies of interventions designed to strengthen parent-teen sexual health communication suggest that ongoing, quality conversations characterized by openness, comfort, and accurate information can increase knowledge about puberty, promote health decision-making skills, and build understanding of the purpose and importance of receiving sexual health care from a qualified clinician.^10,11^

The effectiveness of these conversations, however, may depend on various factors, including the extent of parents’ knowledge and comfort discussing sexual health topics. Parents who are ill equipped to discuss sexual health with their teens may initiate harmful conversations that are associated with increased risk behavior.^12^ Conversely, parents who feel comfortable and ready to discuss intimacy and sexuality, and who demonstrate openness and nonjudgment in their communication, may provide better support and successfully empower their teens to make healthier decisions around sex and bodily autonomy.^13,14^ However, these factors have not been explored at length in research on associations between sexual communication and teen health outcomes. In addition, sexual communication may differ by sociodemographic characteristics of both parents and teens (eg, age, race and ethnicity, sex).^15,16,17^ Understanding the association of these factors with teens’ self-efficacy to seek SRH information and services is necessary to identify potential disparities and work to address them through tailored initiatives designed to support equitable and effective communication.

The present study aims to investigate the association of the frequency of parent-teen sexual health communication frequency with teens’ self-efficacy to seek SRH information and services in a nationally representative sample of US parents and teens. Self-efficacy (ie, one’s belief in their ability to do something) is a proximal antecedent to health behavior change.^18^ Previous research demonstrates connections between communication and self-efficacy,^19,20^ including information-seeking self-efficacy.^21^ Although existing research addresses teen self-efficacy for sexual behaviors such as condom use and sexual communication,^4^ teen self-efficacy to seek SRH information and services is an underexplored area. We explore 2 potential moderators: parents’ perceived information adequacy and comfort regarding sexual health discussions. We also examine whether parents’ sexual health communication frequency, information adequacy, and comfort differ by a number of sociodemographic variables: parent sex, age, race and ethnicity, educational level, and household income, as well as teen sex and sexual orientation.

## Method

Data were collected from parent-teen (aged 15-17 years) dyads participating in the Teen and Parent Surveys of Health. Participants were recruited from AmeriSpeak and AmeriSpeak Teen panels,^22^ which are operated by the National Opinion Research Center (NORC), an independent, nonpartisan research and public policy organization at the University of Chicago.^23^ Panels were designed using probability sampling to be representative of US households. Panel recruitment and management methods are published elsewhere.^23,24^ Of the 2444 invited panelists who completed the screener to determine eligibility for the parent survey, 1230 were determined eligible, and 902 completed informed written consent and were invited to take the survey. Of these, 777 parents completed the survey (June 16 to September 29, 2022; 86.1% response rate). Among teens aged 15 to 17 years, 766 received informed written parental consent and were invited to take the teen survey. Of these, 522 teens completed the survey (May 12 to September 30, 2022; 68.1% response rate), resulting in 522 dyadic pairs. All participants received a cash equivalent of $20 for survey completion; parents also received the equivalent of $2 for completing the screener and consent. This study was approved by NORC’s institutional review board and was consistent with applicable federal law and Centers for Disease Control and Prevention (CDC) policy. The study was prepared in accordance with the Strengthening the Reporting of Observational Studies in Epidemiology (STROBE) reporting guideline for cross-sectional studies.^25^

### Measures

Parents self-reported sex, age, race and ethnicity (Black, non-Hispanic; Hispanic; White, non-Hispanic; and other [participants who reported being multiracial or another race not listed, and were not Hispanic]), educational level, and household income. Race and ethnicity data were collected as part of the standard demographic information gathered from all participants. Teens self-reported sex and sexual orientation. These demographic characteristics were standard questions that all AmeriSpeak panelists receive when they are sampled into the survey. Parents self-reported sexual health communication frequency, information adequacy, and comfort level, and teens self-reported self-efficacy to seek SRH information and services. eTable 1 in Supplement 1 includes item wording and measure operationalization.

#### Parent-Teen Sexual Health Communication Frequency

The original dataset contained 17 parent-teen communication items with the stem “During the past 12 months, how often have you talked with [child’s name] about each of the following?” Exploratory factor analysis was performed to seek the latent structure underlying parent communication frequency items.^26^ The 17 items were summarized into 2 latent scales: “sexual health communication” (eg, “Where to get healthcare services for sexual and reproductive health, like birth control or sexually transmitted infection testing”) and “other communication” (“How to keep information private while using the internet”). Because 2 items showed relatively lower factor loadings, we removed these and reran the factor analysis. Both factor analyses are reported in eTable 2 in Supplement 1. The resulting 7-item parent-teen sexual health communication frequency scale showed evidence of strong reliability (Cronbach α = 0.93; eTable 3 in Supplement 1).^27^ The factor score was extracted as a standardized normal (mean [SD], 0 [1]) and used for the analyses. Items included: (1) [Your child]’s decisions about whether to have sex; (2) How to create and maintain healthy, respectful romantic relationships; (3) The importance of giving and receiving consent for sex; (4) How to say no to sex; (5) How to prevent pregnancy; (6) How to prevent sexually transmitted infections, including HIV; and (7) Where to get health care services for sexual and reproductive health, like birth control or sexually transmitted infection testing. Parents rated their frequency for each item on a 4-point scale: (1 = more than a few times, 2 = a few times, 3 = once or twice, 4 = never). Items were reverse coded, with higher scores indicating that parents communicate more frequently. eTable 4 in Supplement 1 shows associations between parent demographics and individual communication frequency items.

#### Parent Information Adequacy

Parents were asked, “How much do you agree or disagree with the following statement?” with the item: “I have the information I need to talk to [child name] about sex.” Parents responded on a 5-point Likert scale (1 = strongly agree, 5 = strongly disagree). The item was reverse coded, with higher scores indicating parents feeling more informed.

#### Parent Comfort Level

Parents were asked, “How much do you agree or disagree with the following statement?” with the item: “I am comfortable talking to [child name] about sex.” Parents responded on a 5-point Likert scale (1 = strongly agree, 5 = strongly disagree). The item was reverse coded, with higher scores indicating parents feeling more comfortable.

#### Teen Self-Efficacy to Seek SRH Information and Services

Teens were asked, “How much do you agree or disagree with the following statements?” with 5 items (eTable 1 in Supplement 1). Items included (1) “I am confident that I know where to seek information about sexual and reproductive health”; (2) “I am confident using the internet or telephone to find information about sexual and reproductive health”; (3) “I am confident talking with a healthcare provider, like a doctor or nurse, to get information about sexual and reproductive health”; (4) “I have access to resources (eg, provider, internet, friends) about sexual and reproductive health”; and (5) “I am confident sharing information about sexual and reproductive health with a healthcare provider through virtual platforms (eg, a video call).” Items were rated on a 5-point Likert scale (1 = strongly agree, 5 = strongly disagree), and reverse coded, with higher scores indicating higher self-efficacy. Items were averaged for a total scale score.

### Statistical Analysis

Statistical analysis was performed from February to May 2024. The χ^2^ tests assessed whether levels of parent sexual health communication frequency, information adequacy, and comfort level differed by any of our categorical parent-reported and teen-reported demographic variables. For only these analyses, sexual health communication frequency, information adequacy, and communication comfort were treated as categorical to capture demographic group differences among participants (categorization of these variables is presented in the footnotes of Table 1). Univariate and multivariate regression models for parent-reported communication variables and teen self-efficacy were performed. Moderated linear regression analyses evaluated the association between parent sexual health communication frequency and teen self-efficacy to seek SRH information and services, examining the moderating association of 2 prespecified parent variables: information adequacy and communication comfort. Analyses were conducted in Stata, version 18 (StataCorp LLC). All analyses were weighted, and all tests were 2-sided, with statistical significance set at *P* < .05.

**Table 1.** Demographic Characteristics of Parent and Teen Sample by Sexual Health Communication Characteristics, Teen and Parent Surveys of Health (522 Parent-Teen Dyads)

Characteristic	Overall, unweighted No. (% weighted)^a^	Sexual health communication frequency^b^			Perceived sexual health information adequacy^c^				Sexual health communication comfort^d^			
		% (95% CI)		*P* value^e^	% (95% CI)			*P* value^e^	% (95% CI)			*P* value^e^
		Low (≤median)	High (>median)		Inadequate	Ambivalent	Adequate		Discomfort	Ambivalent	Comfort	
**Parent demographic characteristics**												
Sex												
Male	145 (38.7)	66.0 (56.3-74.5)	34.0 (25.5-43.7)	.001	3.6 (1.4-9.1)	18.2 (10.7-29.2)	78.2 (68.9-85.3)	.58	12 (7.7-18.2)	19.6 (14.0-26.7)	68.4 (60.0-75.8)	.08
Female	377 (61.3)	45.5 (39.8-51.4)	54.5 (48.6-60.2)		2.1 (0.7-5.5)	15.1 (10.6-21.3)	82.8 (76.4-87.7)		5.6 (3.7-8.4)	18.5 (12.6-26.2)	76.0 (68.1-82.4)	
Age, y												
18-44	308 (46.5)	42.0 (35.6-48.7)	58.0 (51.3-64.4)	<.001	3.9 (1.7-8.6)	16.3 (11.9-21.9)	79.8 (73.7-84.8)	.48	6.5 (4.0-10.3)	17.3 (13.0-22.7)	76.2 (70.3-81.2)	.37
≥45	214 (53.5)	63.0 (55.8-69.7)	37.0 (30.3-44.2)		1.6 (0.4-5.5)	16.4 (10.1-25.5)	82.0 (74.0-88.0)		9.4 (6.1-14.3)	20.2 (13.8-28.7)	70.3 (61.6-77.8)	
Race and ethnicity												
Black, non-Hispanic	80 (12.8)	25.7 (13.4-43.5)	74.3 (56.5-86.6)	.01	4.6 (0.7-23.7)	26.8 (15.3-42.8)	68.6 (59.0-76.8)	.05	1.5 (0.2-10.3)	29.8 (21.0-40.3)	72.3 (65.1-78.6)	.09
Hispanic	92 (22.7)	47.6 (35.1-60.4)	52.4 (39.6-64.9)		3.7 (0.8-15.4)	21.1 (11.0-36.8)	75.2 (59.2-86.4)		5.5 (2.1-14.0)	17.9 (8.8-33.2)	68.8 (58.1-77.7)	
White, non-Hispanic	309 (57.0)	60.4 (52.7-67.6)	39.6 (32.4-47.3)		1.0 (0.3-2.8)	12.7 (8.8-18.0)	86.3 (81.0-90.3)		9.2 (6.3-13.3)	18.5 (13.4-25.0)	76.5 (61.1-87.1)	
Other^f^	41 (7.5)	61.8 (37.6-81.4)	38.2 (18.6-62.4)		9.2 (3.7-21.3)	11.6 (4.7-25.9)	79.2 (64.9-88.7)		17.8 (7.5-36.6)	7.6 (2.2-23.0)	74.6 (56.8-86.7)	
Educational level												
Less than Bachelor’s degree	325 (56.7)	47.2 (40.4-54.2)	52.8 (45.8-59.6)	.03	2.7 (1.1-6.5)	17.9 (12.4-25.0)	79.4 (72.5-84.9)	.39	6.1 (3.7-9.9)	19.2 (13.6-26.5)	74.7 (66.9-81.2)	.35
Bachelor’s degree or higher	197 (43.3)	61.4 (52.1-70.0)	38.6 (30.0-47.9)		2.6 (0.9-7.3)	14.3 (8.6-23.0)	83.1 (75.9-88.5)		10.7 (7.0-15.9)	18.4 (12.0-27.3)	70.9 (62.4-78.1)	
Household income, $												
<30 000	124 (22.3)	35.5 (27.1-44.9)	64.5 (55.1-72.9)	<.001	4.3 (1.2-14.3)	20.0 (12.7-30.1)	75.7 (64.6-84.2)	.51	2.6 (0.6-9.7)	22.2 (13.8-33.6)	75.3 (63.5-84.2)	.07
30 000 to <60 000	154 (24.9)	46.6 (36.5-56.9)	53.4 (43.1-63.5)		1.9 (0.5-7.2)	17.5 (10.1-28.5)	80.7 (70.0-88.2)		3.7 (1.4-9.3)	19.1 (12.3-28.4)	77.2 (67.2-84.9)	
60 000 to <100 000	121 (24.8)	58.0 (46.4-68.7)	42.0 (31.3-53.6)		1.0 (0.3-3.7)	14.6 (7.9-25.5)	84.4 (73.8-91.2)		10.9 (6.2-18.4)	13.7 (7.0-25.2)	75.3 (63.8-84.1)	
≥100 000	123 (28.1)	68.9 (58.9-77.4)	31.1 (22.6-41.1)		3.6 (1.1-10.9)	13.9 (8.4-22.3)	82.5 (74.8-88.2)		13.7 (8.3-21.8)	20.7 (12.5-32.4)	65.6 (54.8-74.9)	
**Teen demographic characteristics**												
Sex												
Male	244 (50.3)	49.5 (41.5-57.6)	50.5 (42.4-58.5)	.11	1.8 (0.6-6.0)	13.8 (8.5-21.7)	84.3 (77.6-89.3)	.29	6.8 (3.9-11.6)	15.8 (10.7-22.7)	77.4 (70.2-83.3)	.11
Female	273 (49.7)	57.9 (52.4-63.3)	42.1 (36.7-47.6)		3.1 (1.3-7.3)	19.0 (14.2-24.9)	77.9 (71.9-82.8)		9.2 (6.3-13.2)	22.2 (16.8-28.8)	68.6 (62.1-74.4)	
Sexual orientation^g^												
Heterosexual	394 (79.8)	50.4 (44.5-56.2)	49.6 (43.8-55.5)	.09	2.3 (1.0-5.1)	16.9 (12.5-22.5)	80.8 (75.0-85.6)	.52	7.3 (5.0-10.6)	19.9 (14.9-26.0)	72.8 (66.3-78.4)	.43
LGB+	103 (20.2)	59.1 (49.3-68.2)	40.9 (31.8-50.7)		0.8 (0.2-2.7)	18.9 (10.4-31.8)	80.4 (67.6-88.9)		11.7 (5.9-22.0)	15.3 (7.4-29.1)	73.0 (59.5-83.2)	

^a^ Missing responses were treated as “did not report” and included in the denominator.

^b^ Scores were dichotomized: a high score was greater than the median score of 0.0038; a low score was less than or equal to the median score of 0.0038.

^c^ Responses were categorized into 3 levels. Adequate = strongly agree (1) and agree (2); ambivalent = neither agree or disagree (3); and inadequate = disagree (4) and strongly disagree (5).

^d^ Responses were categorized into 3 levels. Comfortable = strongly agree (1) and agree (2); ambivalent = neither agree or disagree (3); and uncomfortable = disagree (4) and strongly disagree (5).

^e^ Determined by use of the χ^2^ test.

^f^ Includes participants who reported being multiracial or another race not listed and were not Hispanic.

^g^ Those who responded “lesbian,” “gay,” “bisexual,” or “something else” were categorized as lesbian, gay, bisexual, or another nonheterosexual orientation (LGB+). A total of 25 respondents indicated “I don’t know the answer” for the sexual orientation question.

## Results

Table 1 displays demographic characteristics of the 522 parents and teens. Parents included 377 women (61.3%) and 145 men (38.7%), were mostly 45 years or older (214 [53.5%]), and identified as non-Hispanic Black (80 [12.8%]), Hispanic (92 [22.7%]), non-Hispanic White (309 [57.0%]), and other race or ethnicity (41 [7.5%]). In terms of educational level, more than half of parents held less than a bachelor’s degree (325 [56.7%]). Over one-fourth of parents reported a household income of $100 000 or more (123 [28.1%]), with the rest distributed across lower income brackets. Among teens, the sample was evenly split by sex (244 male [50.3%] and 273 female [49.7%]), and the majority identified as heterosexual (394 [79.8%]). Approximately 1 in 5 teens (103 [20.2%]) identified as lesbian, gay, bisexual, or another nonheterosexual orientation.

Findings indicated no statistically significant differences in the frequency of parent-reported sexual health communication based on any demographic characteristics of teens; however, frequency differed significantly by parent demographic characteristics (sex, age, race and ethnicity, educational level, and household income). Specifically, female parents had greater likelihood of high-frequency sexual health communication compared with male parents (54.5% [95% CI, 48.6%-60.2%] vs 34.0% [95% CI, 25.5%-43.7%]; *P* = .001) and parents who were younger (18-44 years) were more likely to communicate frequently compared with parents who were 45 years of age or older (58.0% [95% CI, 51.3%-64.4%] vs 37.0% [95% CI, 30.3%-44.2%]; *P* < .001) (Table 1). Black parents reported the most frequent communication (74.3% [95% CI, 56.5%-86.6%]) compared with other racial and ethnic groups (Hispanic parents, 52.4% [95% CI, 39.6%-64.9%]; White parents, 39.6% [95% CI, 32.4%-47.3%]; and parents who were multiracial or another race, 38.2% [95% CI, 18.6%-62.4%]; *P* = .01). Parents who held a bachelor’s degree or higher were less likely to have high sexual health communication frequency compared with parents who had less than a bachelor’s degree (38.6% [95% CI, 30.0%-47.9%] vs 52.8% [95% CI, 45.8%-59.6%]; *P* = .03). Parents with annual household incomes under $30 000 displayed a significantly higher likelihood of frequent sexual health communication (64.5% [95% CI, 55.1%-72.9%]), while parents with incomes of $100 000 or more had the lowest likelihood of communicating (31.1% [95% CI, 22.6%-41.1%]; *P* < .001). There were no statistically significant differences in parents’ information adequacy or comfort level based on parent or teen demographic characteristics.

Table 2 shows the results of both univariate regression models and main effects models with teen self-efficacy as the outcome. In univariate models, communication frequency (β = 0.12 [95% CI, 0.04-0.20]; *P* < .001), information adequacy (β = 0.16 [95% CI, 0.06-0.26]; *P* < .001), and communication comfort (β = 0.11 [95% CI, 0.05-0.18]; *P* < .001) were each positively associated with teen self-efficacy. In main effects models, information adequacy (β = 0.13 [95% CI, 0.01-0.24]; *P* = .03) and communication comfort (β = 0.09 [95% CI, 0.01-0.17]; *P* = .03) remained significant, while communication frequency no longer showed a significant association.

**Table 2.** Univariate and Multivariate Regression Models for Parent-Reported Sexual Health Communication Variables and Teen Self-Efficacy to Seek Sexual Health Services, Teen and Parent Surveys of Health (522 Parent-Teen Dyads)

Model	β (95% CI)	*P* value
**Univariate analysis**		
Model 1		
Intercept	3.83 (3.75 to 3.92)	<.001
Communication frequency	0.12 (0.04 to 0.20)	<.001
Model 2		
Intercept	3.14 (2.69 to 3.59)	<.001
Information adequacy	0.16 (0.06 to 0.26)	<.001
Model 3		
Intercept	3.35 (3.07 to 3.64)	<.001
Communication comfort level	0.11 (0.05 to 0.18)	<.001
**Main effects model**		
Model 1		
Intercept	3.29 (2.80 to 3.78)	<.001
Communication frequency	0.08 (0.00 to 0.17)	.05
Information adequacy	0.13 (0.01 to 0.24)	.03
Model 2		
Intercept	3.47 (3.15 to 3.79)	<.001
Communication frequency	0.08 (−0.01 to 0.16)	.09
Communication comfort level	0.09 (0.01 to 0.17)	.03

**Figure 1.**
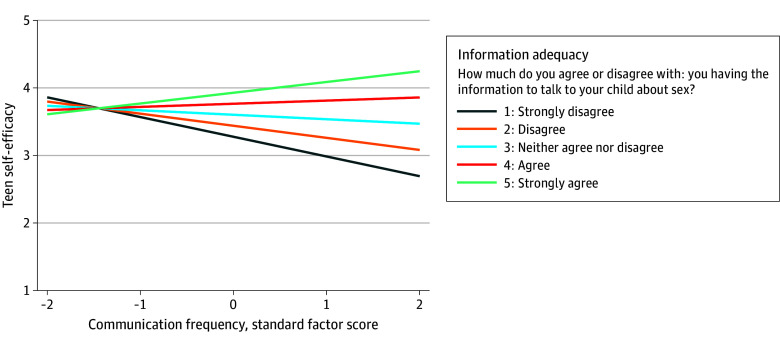
Teens’ Self-Efficacy to Seek Sexual and Reproductive Health (SRH) Services and Parents’ Frequency of Communication About SRH, Moderated by Parent-Perceived Sexual Health Information Adequacy Moderation effects of sexual health information adequacy on the association between parent-teen sexual health communication frequency and teens’ self-efficacy to seek SRH information and services. Sexual health communication frequency was derived from a factor analysis of parent-rated items (see eTable 1 in Supplement 1 for information on operationalization and coding). Higher scores indicate more frequent communication. See the Measures subsection of the Methods section for more detail regarding the teen self-efficacy to seek SRH information and services measure.

**Figure 2.**
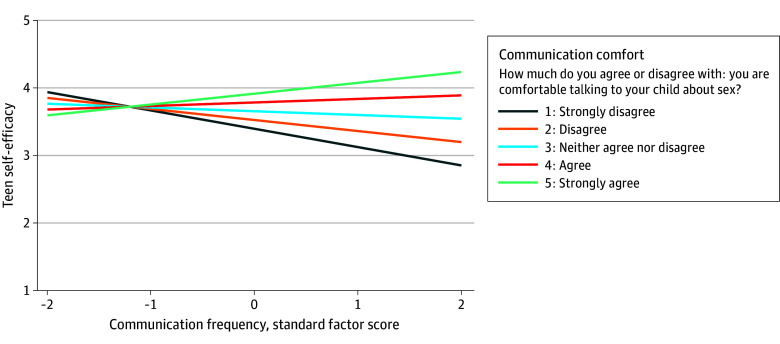
Teens’ Self-Efficacy to Seek Sexual and Reproductive Health (SRH) Services and the Frequency of Parents’ Communication About SRH, Moderated by Parent Report of Sexual Health Communication Comfort Moderation effects of parents’ comfort with communicating with their teen about sex on the association between parent-teen sexual health communication frequency and teens’ self-efficacy to seek SRH information and services. Sexual health communication frequency was derived from a factor analysis of parent-rated items (see eTable 1 in Supplement 1 for information on operationalization and coding). Higher scores indicate more frequent communication. See the Measures subsection of the Methods section for more detail regarding the teen self-efficacy to seek SRH information and services measure.

Table 3 builds on these findings by showing the associations between parent communication frequency and teen self-efficacy, with communication comfort and information adequacy included as moderators to examine interactions. The association was significantly moderated by both variables; teens whose parents talked frequently had higher self-efficacy to seek SRH information and services when parents felt more informed about the topics (β = 0.11 [95% CI, 0.03-0.20]; *P* = .01) (Figure 1 and Table 3), and when parents felt more comfortable communicating (β = 0.11 [95% CI, 0.01-0.20]; *P* = .03) (Figure 2 and Table 3). Slope differences in Figure 1 and Figure 2 indicate that information adequacy and communication comfort each strengthen the positive association between frequent communication and teen self-efficacy to seek SRH information and services. The pattern of results remained the same when adjusting for demographic differences in communication frequency (eTable 5 in Supplement 1).

**Table 3.** Association of the Frequency of Parent-Teen Sexual Health Communication With Teens’ Self-Efficacy to Seek Sexual and Reproductive Health Services, Moderated by Parents’ Perceived Sexual Health Information Adequacy and Communication Comfort (522 Parent-Teen Dyads)

Model	β (95% CI)	*P* value
**Model 1: information adequacy**		
Intercept	3.11 (2.65 to 3.58)	<.001
Sexual health communication frequency	−0.40 (−0.79 to −0.02)	.04
Sexual health information adequacy	0.16 (0.06 to 0.27)	<.001
Sexual health communication frequency × sexual health information adequacy	0.11 (0.03 to 0.20)	.01
**Model 2: communication comfort**		
Intercept	3.26 (2.86 to 3.66)	<.001
Sexual health communication frequency	−0.38 (−0.80 to 0.04)	.08
Sexual health communication comfort	0.13 (0.04 to 0.22)	.01
Sexual health communication frequency × sexual health communication comfort	0.11 (0.01 to 0.20)	.03

## Discussion

Parent-teen sexual health communication can be influential in reducing sexual risk behaviors and helping ensure teens are equipped to navigate sexual and reproductive health care systems.^1,3,26^ This study supplements existing literature on frequent parent-teen sexual communication^1,3,4^ by examining associations between parent communication frequency and teen service-seeking self-efficacy, and the relevance of parents’ comfort and information adequacy. Findings indicate that teens whose parents talk frequently about sexual health generally have higher self-efficacy in seeking services related to sexual health, but this association depends on how comfortable and informed parents feel about the topics. This frequent communication can have a positive influence on teens’ confidence to engage in health-supportive behaviors, but if parents do not feel informed or comfortable regarding sexual health topics, then their more frequent communication is associated with lower teen self-efficacy to seek SRH information and services when needed.

As communication about sexual health topics is not uniformly associated with positive teen health outcomes, simply encouraging parents to “talk about sex more” with their teens may not be sufficient. Rather, the present study’s findings highlight a need for interventions that provide parents with knowledge, skills, and support they will need to feel ready to engage in these conversations. Previous research shows that parents may not feel adequately supported to talk with their teens about sexual health or protection against risky sexual behaviors.^28,29^ Programs designed to increase parents’ comfort around talking about sex with their teens and ensure they have access to current, evidence-based sexual health information may help their communication achieve its intended benefits. School-based, health care professional–based, and community-based interventions can involve parents, focusing on enhancing their knowledge about SRH topics and their ability to discuss them.^3,28,30^ Similar interventions have been shown to improve teens’ feelings of parental support and have resulted in fewer behavioral risks.^31^ The CDC’s Division of Adolescent and School Health offers resources for parents, including CDC Healthy Youth Parent Resources and tips for Talking with Your Teen about Sex.^32,33^ Programs with structured workshops or online modules offering a judgment-free setting and concrete strategies for initiating and maintaining open dialogue about SRH topics may increase parents’ knowledge and comfort communicating, although further research is needed to understand how best to implement such interventions.^34,35,36^

Consistent with previous research, mothers (compared with fathers) had a greater likelihood of engaging in sexual health discussions with their teens,^17,28,37,38^ a pattern that may reflect broader norms around gender socialization, where mothers are expected to play a more active role in their children’s upbringing, including providing sexual health education.^39,40,41,42^ Black parents also indicated more frequent discussion about sexual health compared with parents of other racial and ethnic groups. Previous research studies suggest this pattern may be indicative of greater awareness of (and desire to combat) sexual health disparities within Black communities, where Black teens face disproportionately higher rates of HIV or sexually transmitted infections and unintended pregnancies due to structural inequities (eg, limited availability of affordable health care; implicit racial bias among health care professionals).^43,44,45,46^ As one study shows, early and frequent parent-teen sexual health communication is a protective strategy used among Black families to delay sexual initiation and promote safer sexual behavior.^47^ However, there was cultural variability; for example, Haitian families (compared with African American and Jamaican families) expressed lower comfort, value, and breadth of parent-teen sexual health communication.^47^ This finding speaks to the necessity of not treating Black families as a monolith—in general, but particularly in this kind of research. Interventions should be culturally sensitive and tailored to families’ comfort levels and values surrounding sex.^48,49,50^ In addition, because studies have shown that parents of racial and ethnic minority groups report lower effectiveness beliefs concerning their sexual health communication, future research should seek to identify specific barriers that parents of racial and ethnic minority groups face and work to eliminate these barriers.^28,51,52^

Our study also found that parents with lower educational levels and annual incomes discussed sexual health topics more frequently; this may result from attempts to fill gaps in their teens’ education if the families do not have access to high-quality sex education programs. Younger parents (<45 years) also reported more frequent communication, potentially indicating a shift in cultural norms surrounding open sexual communication. Together, these findings illustrate factors shaping parent-teen communication dynamics, and prompt a need for more exploration of these factors, how they intersect, and how we could design and deliver interventions to promote effective, equitable sexual health communication within families. Studies may also consider teens’ perceptions more in depth, as factors such as teens’ trust in their parents and comfort discussing sex with them may also be associated with the effectiveness of these conversations.

### Limitations and Strengths

This study has several limitations. First, while our sample was designed to be representative of US households, the surveys were offered only in English, and parental consent was required for teen participation, which may have led to sampling bias or exclusion.^53,54^ Second, the small sample size limited analyses among certain demographic subgroups, possibly resulting in suppression of differences. Even so, previous studies of parent-teen sexual communication have relied on convenience or nonrandom samples,^55^ so our use of a probability-based sample may strengthen generalizability of findings. Third, sexual health communication frequency reported by parents may be influenced by social desirability bias and subsequently result in underestimation or overestimation of communication. We must also consider the possibility of overlap in participants’ interpretations of the response options for this measure. For example, a parent who discussed a sexual health topic 4 to 6 times within the past year may have considered this “a few times,” while another parent with the same actual communication frequency considered this “more than a few times,” resulting in potential variability within response categories. Fourth, our outcome measure focused on teens’ self-efficacy to seek SRH information and services, rather than the act of service seeking. Despite theoretical and empirical evidence positioning self-efficacy as a key factor associated with health behavior,^56,57,58,59^ our findings do not speak to whether teens engaged in that behavior. In other words, even if teens feel confident in their ability to seek health services, there may be other barriers and situational factors preventing them from doing so when necessary.^60^ Fifth, our parent-perceived information adequacy variable relies on self-reported adequacy, which may or may not accurately reflect the actual accuracy of the information parents have. Despite these limitations, to our knowledge, this cross-sectional study is the first to assess parents’ perceived information adequacy and comfort with sexual health communication as moderators for the association between the frequency of parent-teen sexual health communication and teen-reported self-efficacy to seek SRH information and services. The study’s dyadic nature helped ensure incorporation of both parent and teen perspectives and can strengthen development of interventions that aim to optimize teens’ health and quality of life.^61^

## Conclusions

In this cross-sectional study, the frequency with which parents spoke to their children about sex and sexual health was associated with teens’ self-efficacy to seek sexual health information and services, as long as parents were informed and comfortable talking about these topics. Our findings suggest that parents need to have quality information about sexual health and effective strategies to increase their comfort in having these discussions.

## Notes

Supplement 1. **eTable 1.** Measurement Dictionary for Key Variables of Interest From the Teen and Parent Surveys of Health, 2022 **eTable 2.** Exploratory Factor Analyses **eTable 3.** Evidence of Validity and Reliability for Survey Items Comprising Sexual Health Communication Frequency Construct **eTable 4.** Associations Between Parent Demographic Characteristics and Individual Survey Items Comprising Sexual Health Communication Frequency Construct **eTable 5.** Main Results Adjusting for Demographic Covariates

Supplement 2.
Data Sharing Statement
