# Supplementary material for: Parent-Teen Sexual Health Communication and Teens’ Health Information and Service Seeking
Source: JAMA Netw Open. 2025 Nov 5;8(11):e2541712. doi: 10.1001/jamanetworkopen.2025.41712 (PMC12590298; doi:10.1001/jamanetworkopen.2025.41712)
Supplement: Supplement 1. — eTable 1. Measurement Dictionary for Key Variables of Interest From the Teen and Parent Surveys of Health, 2022 eTable 2. Exploratory Factor Analyses eTable 3. Evidence of Validity and Reliability for Survey Items Comprising Sexual Health Communication Frequency Construct eTable 4. Associations Between Parent Demographic Characteristics and Individual Survey Items Comprising Sexual Health Communication Frequency Construct eTable 5. Main Results Adjusting for Demographic Covariates [file jamanetwopen-e2541712-s001.pdf]

Supplementary Online Content

Javidi H, Verlenden JV, Chen X, Walsh-Buhi ER. Parent-teen sexual health communication and teens' health information and service seeking. *JAMA Netw Open.* 2025;8(11):e2541712. doi:10.1001/jamanetworkopen.2025.41712

eTable 1. Measurement Dictionary for Key Variables of Interest From the Teen and Parent Surveys of Health, 2022
eTable 2. Exploratory Factor Analyses
eTable 3. Evidence of Validity and Reliability for Survey Items Comprising Sexual Health Communication Frequency Construct
eTable 4. Associations Between Parent Demographic Characteristics and Individual Survey Items Comprising Sexual Health Communication Frequency Construct
eTable 5. Main Results Adjusting for Demographic Covariates

This supplementary material has been provided by the authors to give readers additional information about their work.

eTable 1. Measurement Dictionary for Key Variables of Interest From the Teen and Parent Surveys of Health, 2022

Measures	Survey Items and Response Options	Operationalization
Self-efficacy to seek sexual and reproductive health services (teens, self-report)	Stem: How much do you agree or disagree with the following statements?<br><br>a. I am confident that I know where to seek information about sexual and reproductive health.<br>b. I am confident using the internet or telephone to find information about sexual and reproductive health.<br>c. I am confident talking with a healthcare provider, like a doctor or nurse, to get information about sexual and reproductive health.<br>d. I am confident I have access to resources (e.g., provider, internet, friends) about sexual and reproductive health.<br>e. I am confident sharing information about sexual and reproductive health with a healthcare provider through virtual platforms (e.g., a video call)<br><br>Response Options:<br>1=Strongly agree<br>2=Agree<br>3=Neither agree nor disagree<br>4=Disagree<br>5=Strongly disagree	Responses were reverse-coded. Scores for the 5 items (a-e) were averaged to create an overall average score for self-efficacy to seek sexual and reproductive health services.
Parent-teen sexual and reproductive health communication frequency (parent-report)	During the past 12 months, how often have you talked with [CHILDNAME] about each of the following?<br>a. [CHILDNAME]'s decisions about whether to have sex<br>b. How to create and maintain healthy, respectful romantic relationships<br>c. The importance of giving and receiving consent for sex<br>d. How to say no to sex<br>e. How to prevent pregnancy<br>f. How to prevent sexually transmitted infections (STIs), including HIV<br>g. Where to get healthcare services for sexual and reproductive health, like birth control or STI testing<br><br>Response Options:<br>1=More than a few times<br>2=A few times<br>3=Once or twice<br>4=Never	Responses were reverse-coded. Factor scores for the 7 items were extracted from a factor analysis to create an overall communication frequency score.<br><br>Low frequency ≤ median (0.0038)<br>High frequency > median (0.0038)
Sexual Health Information Adequacy (parent-report)	How much do you agree or disagree with the following statement?<br>I have the information I need to talk to [CHILDNAME] about sex.<br><br>Response Options:<br>1=Strongly agree<br>2=Agree<br>3=Neither agree nor disagree	High = responded 1, 2, or 3<br>Low = responded 4 or 5

	4=Disagree<br>5=Strongly disagree	
Sexual Health Communication Comfort (parent-report)	How much do you agree or disagree with the following statement?<br>I am comfortable talking to [CHILDNAME] about sex.<br><br>Response Options:<br>1=Strongly agree<br>2=Agree<br>3=Neither agree nor disagree<br>4=Disagree<br>5=Strongly disagree	High = responded 1, 2, or 3<br>Low = responded 4 or 5

[a]Surveys were administered in English to parent-teen (age 15-17 years) dyadic pairs participating in the Teen and Parent Surveys of Health (TAPS). Participants were recruited from the AmeriSpeak® and AmeriSpeak Teen panels, which are designed to be representative of U.S. household populations.

© 2025 Javidi H et al. *JAMA Network Open.*

eTable 2. Exploratory Factor Analyses

Factor analysis of all 17 items

Item	Question	Factor 1	Factor 2
Q6A	Treating people [your child] dates respectfully or being treated respectfully	0.467	0.379
Q6B	Sexting	0.560	0.360
Q6C	[Your child]'s decisions about whether to have sex	0.680	0.272
Q6D	How to create and maintain healthy, respectful romantic relationships	0.613	0.413
Q6E	The importance of giving and receiving consent for sex	0.779	0.292
Q6F	How to say no to sex	0.832	0.220
Q6G	How to prevent pregnancy	0.861	0.202
Q6H	How to prevent sexually transmitted infections (STIs), including HIV	0.890	0.174
Q6I	Where to get healthcare services for sexual and reproductive health, like birth control or STI testing	0.820	0.172
Q6J	Where to get help for stress, anxiety, and depression	0.446	0.509
Q6K	Dangers of smoking cigarettes, using e-cigarettes or vaping devices, drinking alcohol, and using marijuana or other drugs	0.360	0.634
Q6L	How to avoid online risks like chatting with strangers	0.407	0.641
Q6M	How to keep information private while using the internet	0.419	0.579
Q6N	[Your child]'s relationships with their friends	0.203	0.663
Q6O	Treating people of different races or ethnicities with respect	0.304	0.615
Q6P	How [your child] is doing in school	0.045	0.559
Q6Q	The range of sexual orientations and gender identities	0.312	0.591
**Cronbach's alpha**		**0.929**	**0.870**

Factor analysis after removing Q6A and Q6B

Item	Question	Factor 1	Factor 2
Q6C	[Your child]'s decisions about whether to have sex	0.635	0.262
Q6D	How to create and maintain healthy, respectful romantic relationships	0.583	0.403
Q6E	The importance of giving and receiving consent for sex	0.777	0.296
Q6F	How to say no to sex	0.838	0.227
Q6G	How to prevent pregnancy	0.868	0.203
Q6H	How to prevent sexually transmitted infections (STIs), including HIV	0.900	0.179
Q6I	Where to get healthcare services for sexual and reproductive health, like birth control or STI testing	0.833	0.181
Q6J	Where to get help for stress, anxiety, and depression	0.444	0.508
Q6K	Dangers of smoking cigarettes, using e-cigarettes or vaping devices, drinking alcohol, and using marijuana or other drugs	0.360	0.633
Q6L	How to avoid online risks like chatting with strangers	0.414	0.646
Q6M	How to keep information private while using the internet	0.429	0.589
Q6N	[Your child]'s relationships with their friends	0.204	0.661
Q6O	Treating people of different races or ethnicities with respect	0.304	0.610
Q6P	How [your child] is doing in school	0.046	0.553
Q6Q	The range of sexual orientations and gender identities	0.312	0.592
**Cronbach's alpha**		**0.931**	**0.870**

© 2025 Javidi H et al. *JAMA Network Open.*

eTable 3. Evidence of Validity and Reliability for Survey Items Comprising Sexual Health Communication Frequency Construct

Survey Items	Factor Loading
**Sexual health communication frequency** (α = 0.93)[a]	
1. [Your child]'s decisions about whether to have sex	0.64
2. How to create and maintain healthy, respectful romantic relationships	0.58
3. The importance of giving and receiving consent for sex	0.78
4. How to say no to sex	0.84
5. How to prevent pregnancy	0.87
6. How to prevent sexually transmitted infections (STIs), including HIV	0.90
7. Where to get healthcare services for sexual and reproductive health, like birth control or STI testing	0.83

[a]Cronbach's alpha

© 2025 Javidi H et al. *JAMA Network Open.*

eTable 4. Associations Between Parent Demographic Characteristics and Individual Survey Items Comprising Sexual Health Communication Frequency Construct

	Parent Sex			Parent Age			Parent Race/Ethnicity				
	Male	Female		18-44 years	45 years and older		White, non-Hispanic	Black, non-Hispanic	Hispanic	Non-Hispanic, multiracial, or another race	
Survey Item	N (%)	N (%)	Chi-square P-value	N (%)	N (%)	Chi-square P-value	N (%)	N (%)	N (%)	N (%)	Chi-square P-value
1. [Your child]'s decisions about whether to have sex			0.01			0.04					0.72
More than a few times	29 (14.56)	92 (28.78)		70 (28.75)	52 (18.51)		69 (23.13)	18 (27.5)	28 (23.67)	6 (15.88)	
Less often or Never	173 (85.44)	228 (71.22)		173 (71.25)	227 (81.49)		229 (76.87)	48 (72.5)	90 (76.33)	33 (84.12)	
2. How to create and maintain healthy, respectful romantic relationships			0.02			0.00					0.42
More than a few times	45 (22.23)	114 (35.6)		92 (37.73)	67 (24.09)		85 (28.43)	28 (41.21)	36 (30.36)	11 (27.45)	
Less often or Never	157 (77.77)	206 (64.4)		151 (62.27)	212 (75.91)		213 (71.57)	39 (58.79)	83 (69.64)	28 (72.55)	
3. The importance of giving and receiving consent for sex			0.00			0.01					0.03
More than a few times	32 (15.71)	101 (31.61)		80 (32.97)	53 (18.95)		69 (23.1)	29 (43.48)	28 (23.95)	7 (17.29)	
Less often or Never	170 (84.29)	219 (68.39)		163 (67.03)	227 (81.05)		229 (76.9)	38 (56.52)	90 (76.05)	32 (82.71)	
4. How to say no to sex			<0.001			0.01					0.00
More than a few times	18 (9.07)	105 (32.84)		74 (30.81)	49 (17.43)		57 (19.14)	33 (48.98)	25 (21.28)	8 (21.34)	
Less often or Never	184 (90.93)	214 (67.16)		167 (69.19)	231 (82.57)		239 (80.86)	34 (51.02)	93 (78.72)	31 (78.66)	
5. How to prevent pregnancy			<0.001			0.004					0.03
More than a few times	30 (14.9)	121 (37.86)		91 (37.69)	60 (21.38)		72 (24.34)	33 (50.01)	32 (26.74)	14 (35.43)	
Less often or Never	172 (85.1)	199 (62.14)		151 (62.31)	219 (78.62)		225 (75.66)	33 (49.99)	87 (73.26)	25 (64.57)	
6. How to prevent sexually transmitted infections (STIs), including HIV			<0.001			<0.001					0.01
More than a few times	31 (15.48)	120 (37.65)		96 (39.88)	55 (19.81)		68 (23.09)	35 (53.03)	37 (31.49)	10 (26.72)	
Less often or Never	169 (84.52)	199 (62.35)		145 (60.12)	223 (80.19)		228 (76.91)	31 (46.97)	81 (68.51)	28 (73.28)	
7. Where to get healthcare services for sexual and reproductive health, like			<0.001			<0.001					0.01

© 2025 Javidi H et al. *JAMA Network Open.*

birth control or STI testing										
More than a few times	17 (8.68)	94 (29.51)		72 (30.02)	39 (14.14)		51 (16.97)	28 (42.76)	27 (22.73)	6 (16.41)
Less often or Never	182 (91.32)	225 (70.49)		168 (69.98)	239 (85.86)		247 (83.03)	37 (57.24)	90 (77.27)	32 (83.59)

	**Parent Education**			**Teen Sex**			**Teen Sexual Orientation**		
	**Less than a Bachelor's degree**	**Bachelor's degree or higher**		**Male**	**Female**		**Heterosexual**	**LGB+**	
	**N (%)**	**N (%)**	**Chi-square P-value**	**N (%)**	**N (%)**	**Chi-square P-value**	**N (%)**	**N (%)**	**Chi-square P-value**
1. [Your child]'s decisions about whether to have sex			0.57			0.35			0.18
More than a few times	72 (24.36)	49 (21.85)		55 (20.97)	65 (25.15)		87 (22.08)	30 (29.82)	
Less often or Never	223 (75.64)	177 (78.15)		206 (79.03)	192 (74.85)		306 (77.92)	70 (70.18)	
2. How to create and maintain healthy, respectful romantic relationships			0.49			0.95			0.55
More than a few times	86 (29.01)	73 (32.29)		78 (30.04)	78 (30.31)		116 (29.44)	34 (33.75)	
Less often or Never	210 (70.99)	153 (67.71)		182 (69.96)	179 (69.69)		278 (70.56)	66 (66.25)	
3. The importance of giving and receiving consent for sex			0.31			0.10			0.18
More than a few times	82 (27.75)	51 (22.47)		74 (28.56)	56 (21.56)		108 (27.33)	21 (20.64)	
Less often or Never	214 (72.25)	175 (77.53)		186 (71.44)	202 (78.44)		286 (72.67)	79 (79.36)	
4. How to say no to sex			0.01			0.33			0.53
More than a few times	86 (29.18)	37 (16.29)		55 (21.09)	65 (25.54)		96 (24.34)	21 (21.01)	
Less often or Never	210 (70.82)	188 (83.71)		205 (78.91)	191 (74.46)		298 (75.66)	78 (78.99)	
5. How to prevent pregnancy			<0.001			0.70			0.11
More than a few times	105 (35.47)	46 (20.43)		77 (29.46)	71 (27.76)		123 (31.24)	24 (23.61)	
Less often or Never	191 (64.53)	179 (79.57)		184 (70.54)	186 (72.24)		270 (68.76)	76 (76.39)	
6. How to prevent sexually transmitted infections (STIs), including HIV			<0.001			0.84			0.08
More than a few times	115 (38.9)	36 (16.25)		77 (29.64)	73 (28.73)		123 (31.42)	23 (23.32)	
Less often or Never	180 (61.1)	188 (83.75)		183 (70.36)	182 (71.27)		269 (68.58)	76 (76.68)	
7. Where to get healthcare services for sexual and			0.00			0.91			0.13

© 2025 Javidi H et al. *JAMA Network Open.*

reproductive health, like birth control or STI testing								
More than a few times	80 (27.42)	31 (13.76)		55 (21.23)	56 (21.75)		93 (23.68)	16 (16.79)
Less often or Never	213 (72.58)	194 (86.24)		203 (78.77)	201 (78.25)		299 (76.32)	81 (83.21)

Note. Responses were collapsed to evaluate the associations between demographic characteristics and individual parent-teen sexual health communication, with “more than a few times” coded as 1 (frequent communication) and all other response options coded as 0 (infrequent or no communication).

© 2025 Javidi H et al. *JAMA Network Open.*

eTable 5. Main Results Adjusting for Demographic Covariates

	Coefficient (95% CI)	P-value
**Model 1: Main effects, moderation, and covariates**		
Intercept	3.13 (2.64 – 3.63)	<0.001
Communication frequency	-0.45 (-0.85 – -0.05)	0.03
Information adequacy	0.15 (0.04 – 0.26)	0.01
Communication frequency * Information adequacy	0.13 (0.04 – 0.22)	0.01
Gender		
Male	Ref.	
Female	0.13 (-0.05 – 0.31)	0.14
Age		
18-44 years	Ref.	
45 years and older	0.19 (0.00 – 0.38)	0.05
Race/ethnicity		
White, non-Hispanic	Ref.	
Black, non-Hispanic	-0.01 (-0.21 – 0.19)	0.91
Hispanic	-0.27 (-0.52 – -0.02)	0.04
Non-Hispanic, multiracial, or another race	-0.17 (-0.44 – 0.10)	0.21
Education		
Less than a Bachelor’s degree	Ref.	
Bachelor's degree or higher	0.07 (-0.10 – 0.23)	0.42
Household income		
Less than $30,000	Ref.	
$30,000 to under $60,000	-0.10 (-0.30 – 0.10)	0.32
$60,000 to under $100,000	-0.28 (-0.52 – -0.03)	0.03
$100,000 or more	-0.01 (-0.31 – 0.28)	0.94
**Model 2: Main effects, moderation, and covariates**		
Intercept	3.22 (2.72 – 3.72)	<0.001
Communication frequency	-0.32 (-0.76 – 0.11)	0.15
Communication comfort	0.13 (0.03 – 0.22)	0.01
Communication frequency * Communication comfort	0.10 (0.00 – 0.19)	0.05
Gender		
Male	Ref.	
Female	0.14 (-0.04 – 0.33)	0.12
Age		
18-44 years	Ref.	
45 years and older	0.21 (0.01 – 0.40)	0.04
Race/ethnicity		
White, non-Hispanic	Ref.	
Black, non-Hispanic	-0.04 (-0.28 – 0.19)	0.71
Hispanic	-0.27 (-0.51 – -0.03)	0.03
Non-Hispanic, multiracial, or another race	-0.18 (-0.43 – 0.08)	0.17
Education		
Less than a Bachelor’s degree	Ref.	

© 2025 Javidi H et al. *JAMA Network Open.*

Bachelor's degree or higher	0.06 (-0.10 – 0.22)	0.49
Household income		
Less than $30,000	Ref.	
$30,000 to under $60,000	-0.09 (-0.30 – 0.12)	0.40
$60,000 to under $100,000	-0.27 (-0.53 – -0.02)	0.04
$100,000 or more	0.01 (-0.28 – 0.29)	0.96

© 2025 Javidi H et al. *JAMA Network Open.*
