# Supplementary material for: Parent-Teen Sexual Health Communication and Teens’ Health Information and Service Seeking
Source: JAMA Netw Open. 2025 Nov 5;8(11):e2541712. doi: 10.1001/jamanetworkopen.2025.41712 (PMC12590298; doi:10.1001/jamanetworkopen.2025.41712)
Supplement: Supplement 2. — Data Sharing Statement [file jamanetwopen-e2541712-s002.pdf]

Data Sharing Statement

Javidi. Parent-Teen Sexual Health Communication and Teens' Health Information and Service Seeking. *JAMA Netw Open*. Published November 05, 2025.
doi:10.1001/jamanetworkopen.2025.41712

Data
Data available: No

Additional Information
Explanation for why data not available: Consistent with our ethics approval, the data are stored on secure cloud storage. Researchers who are interested in accessing the data used in this submission can contact us directly for access
